# Is the information of systematic reviews published in nursing journals up-to-date? a cross-sectional study

**DOI:** 10.1186/s12874-017-0432-3

**Published:** 2017-11-25

**Authors:** Wilson W. S. Tam, Kenneth K. H. Lo, Parames Khalechelvam, Joey Seah, Shawn Y. S. Goh

**Affiliations:** 1 Alice Lee Centre for Nursing Studies, Yong Loo Lin School of Medicine, Level 2, Clinical Research Centre, Block MD11, 10 Medical Drive, Singapore, 117597 Singapore; 2 4/F, JC School of Public Health and Primary Care, The Chinese University of Hong Kong, Shatin, HKSAR Hong Kong

**Keywords:** Systematic reviews, Quality of reporting, Information retrieval, Presentation and publication policy, Evidence-based nursing

## Abstract

**Background:**

An up-to-date systematic review is important for researchers to decide whether to embark on new research or continue supporting ongoing studies. The aim of this study is to examine the time taken between the last search, submission, acceptance and publication dates of systematic reviews published in nursing journals.

**Methods:**

Nursing journals indexed in Journal Citation Reports were first identified. Thereafter, systematic reviews published in these journals in 2014 were extracted from three databases. The quality of the systematic reviews were evaluated by the AMSTAR. The last search, submission, acceptance, online publication, full publication dates and other characteristics of the systematic reviews were recorded. The time taken between the five dates was then computed. Descriptive statistics were used to summarize the time differences; non-parametric statistics were used to examine the association between the time taken from the last search and full publication alongside other potential factors, including the funding support, submission during holiday periods, number of records retrieved from database, inclusion of meta-analysis, and quality of the review.

**Results:**

A total of 107 nursing journals were included in this study, from which 1070 articles were identified through the database search. After screening for eligibility, 202 systematic reviews were included in the analysis. The quality of these reviews was low with the median score of 3 out of 11. A total of 172 (85.1%), 72 (35.6%), 153 (75.7%) and 149 (73.8%) systematic reviews provided their last search, submission, acceptance and online published dates respectively. The median numbers of days taken from the last search to acceptance and to full publication were, respectively, 393 (IQR: 212–609) and 669 (427–915) whereas that from submission to full publication was 365 (243–486). Moreover, the median number of days from the last search to submission and from submission to online publication were 167.5 (53.5–427) and 153 (92–212), respectively. No significant association were revealed between the time lag and those potential factors.

**Conclusion:**

The median time from the last search to acceptance for systematic reviews published in nursing journals was 393 days. Readers for systematic reviews are advised to check the time taken from the last search date of the reviews in order to ensure that up-to-date evidence is consulted for effective clinical decision-making.

**Electronic supplementary material:**

The online version of this article (10.1186/s12874-017-0432-3) contains supplementary material, which is available to authorized users.

## Background

A systematic review (SR) refers to secondary research that not only systematically synthesizes all available research evidence relevant to a particular topic, but also interprets, evaluates and appraises the quality of such results [1]. With up-to-date results from SRs, relevant evidence becomes available for researchers to decide not only whether to embark on new research but also whether to continue supporting ongoing studies [2, 3]. Many policymakers and stakeholders seek to use research evidence to influence policymaking, for whom systematic reviews have become an indispensable resources. [4] With the dynamic nature of gathering and presenting research evidence, SRs must be up-to-date to enable different stakeholders such as researchers, funding bodies, and data-monitoring committees, to use reliable evidence to inform decision-making [2].

Against this background, it is therefore important to ascertain whether the information published in the SRs are up-to-date. In their evaluation of 300 SRs published in Core Clinical Journals, Beller et al. [5] found that the median time from the last search date to first publication was 8 months (~240 days). Another study reported that the median time taken for a research article published in top nursing journals from the end of data collection to first publication was 855 days [6]. However, an interesting gap to explore has remained in the literature: no studies have been conducted to specifically examine the time lag for SRs published in nursing journals.

SRs with longer time lags of publication may appear to be less convincing for the stakeholders due to the concern with less updated evidence. However, such impact on decision-making also depends on the frequency of publication of primary studies in the topic. For example, if multiple new studies are published every year, one or 2 years’ delay in publishing the SR will imply the untimely exclusion of such newly published studies; otherwise, there is less impact of delayed publication on the timeliness of findings. A recently published meta-epidemiological study [7] reported that 73% of the SRs published in top medical journals included 10 or more studies while Pollkki et al. [8] reported a median of 15 studies were included among 39 SRs in high-impact nursing journals. Nevertheless, they did not examine the average number of those studies by the years of the SRs.

The time taken for completing the SRs also affects the time required for publication. Problem identification, search-strategy development, database search, study selection, data extraction, data analysis and synthesis are the basic steps in conducting systematic reviews. Intuitively, if copious records are retrieved from database, more time is expected for study selection and data extraction; therefore, the number of records retrieved may relate to the time taken for publication. Furthermore, performing meta-analysis requires specific training but it has been reported that many nursing researchers and students did not fully understand the statistical techniques of meta-analysis or the various important concepts that underpin it [9]. Thus, the inclusion of meta-analyses in the SR may take more time to complete the review.

Another aspect to influence the speed of publication is the journal review process. Publishing a SR in a peer-reviewed journal usually involves several phases including manuscript submission, first decisions from editors, peer-reviews, follow-up decisions from the editors, and revision and re-submission. The whole process can be repeated numerous times if the SR is rejected at the editor’s first decision, thereby prolonging the publication process: it has been suggested that editors of nursing journals would take at least 8 weeks to first decide whether to accept a manuscript, or to request revisions [10]. In addition, the timing of submission has been suggested to affect the time taken for the peer-reviews as it would be difficult for editors to recruit peer-reviewers during holiday periods [11]. No studies have hitherto examined these effects in the publication process of SRs but they are clearly critical.

On the other hand, the methodological quality of SRs plays an important role in drawing conclusions on intervention effectiveness and carries implications for clinical decision-making. [12] Although studies have examined the methodological quality of SRs [8, 13–15], none of them have examined the association between the quality and the time taken for publication. Besides, Gomez-Garcia et al. [16] reported that funding from academic institutions was associated with a higher quality of SRs and hence the funding sources may also affect the time lag.

Given the aforementioned uncertainties, the primary aim of this study is to evaluate the timeliness of SRs published in nursing journals upon first publication, as measured by the time taken from the last search date to actual publication. The total time taken was divided into stages, including the time from the last search dates to the acceptance, online publication, or full publication. The secondary aim is to explore the factors potentially associated with the time taken, such as study quality, funding source, number of submission, et cetera.

## Methods

### Study design

A cross-sectional study was conducted to examine the time taken from the last search date to actual publication of SRs in nursing journals. The SRs were extracted from three scholarly databases in 2014.

### Inclusion and exclusion criteria

Articles included in this study are self-proclaimed SRs, systematic literature reviews or those that included the term “meta-analyses” in the title, abstract, or both. Only SRs in English were included. Methodological papers, commentaries, conference abstracts, or letters on SRs or meta-analyses were excluded.

### Search strategy

Nursing journals were first identified from the category of “Nursing Studies” within the 2013 edition for Journal Citation Reports (Science) as this was the latest available version in early 2015. This approach has been widely used to identify journal titles for a specific field in citation analysis [17, 18]. A search, involving combinations of journal titles and keywords, was then conducted to identify the SRs from three databases, namely, Cumulative Index of Nursing and Allied Health Literature (CINAHL), PubMed, and Web of Science. The keyword search included “systematic review,” “meta*analys*,” or “pooled analysis*”, were adopted to identify SRs and meta-analyses in other studies [19, 20]. Only SRs published in 2014 were included based on their full publication date (with volume and page numbers) as this study was concluded in 2015. The results from each database were then imported to EndNote X7 with duplicate records removed. The final search was conducted in May 2015. The SRs were originally identified for another study [20].

### Selection of articles

The titles and abstracts of the articles were screened by two independent reviewers (KL and PK) to identify the SRs for inclusion. Full texts of all potential articles were then downloaded for further evaluation. Any discrepancies between the two reviewers were resolved by reaching a consensus. When incongruities persisted, a third reviewer (WT) was consulted to confirm the final inclusion.

### Data extraction

Data of the SRs, including the first author’s name, title, journal name, number of pages, inclusion of meta-analysis, funding source, and other information, were extracted by two independent reviewers (WT and PK). WT and SG then classified the SRs according to the four scopes (Clinical Research, Health Systems and Outcomes Research, Nursing Education Research, and Others) as suggested by the American Association of College of Nursing [21]. Dates of full publication, online publication, acceptance and submission of the included SRs were extracted from the databases, journal homepages, or both. If only the month or months, such as March or March to April, were available, then the first date of the period was used as the date (i.e., March 1st). The last search date of each SR was extracted from the full text. If only the year was specified, then the mid-year (i.e., June 1st) was used as the search end date. The differences (in days) between the last search date and (i) publication date, (ii) online publication date, and (iii) acceptance date were computed for each SR. We also contacted the corresponding author of each included SR by e-mail to investigate the number of journals to which he or she submitted before the SR was accepted for publication.

### Quality assessment

The methodological quality of the included SRs was assessed by A Measurement Tool to Assess Systematic Reviews (AMSTAR), a validated checklist comprising 11 items addressing important aspects of SRs such as the availability of protocols, use of independent reviewers, comprehensiveness of databases, exploration of grey literature, and quality assessment of included studies. [12, 22]. Each of the checklist items was scored “Yes”, “No”, “Cannot Answer” or “Not Applicable” (NA). A higher number of items assigned “Yes”, signifies a superior methodological quality of the reviews. The assessment was conducted by two authors independently (WT and JS), who subsequently resolved any discrepancies by consensus. The number of “Yes” for each item was counted for each review. An overall score of 4 or less represented poor methodological quality, 5 to 8 was considered fair to good, and 9 or greater was deemed to be good [23].

### Data analysis

Descriptive statistics, including the median, inter-quartile range (IQR), frequency, and percentages, were used to summarize the characteristics of the included SRs. The median and IQR were then computed for the differences in all included studies. The Mann-Whitney U or Kruskal-Wallis test was used to examine whether the time from last search to full publication varied by different characteristics including the quality of the SRs, availability of funding support, holiday period, and number of records identified in database search. All analyses were conducted using IBM SPSS statistics 24, while the PRISMA flowchart was generated using PRISMA Flow Diagram Generator [24].

## Results

**Fig. 1:**
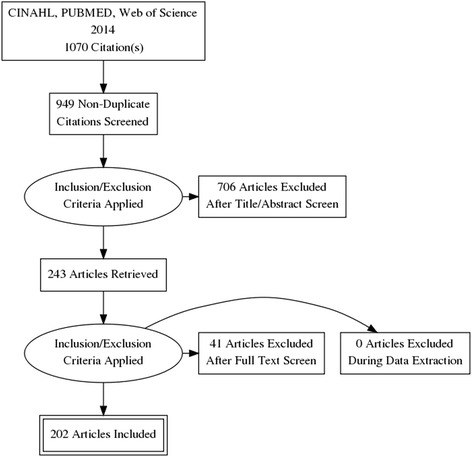
PRISMA flowchart. The diagram was generated using PRISMA Flow Diagram Generator by Toronto Health Economics and Technology Assessment Collaboration (http://prisma.thetacollaborative.ca/)

A total of 107 journals were identified from the Journal Citation Reports (see Additional file 1: Table S1 for the list of journals with the hyperlinks to their homepages), from which 1070 articles were identified during the database search. Upon removal of duplicate articles and those from non-nursing journals (e.g., *Heart and Lung* belonged to nursing journals whereas *Heart, Lung and Circulation* did not), 949 articles were screened and evaluated through their title and abstract for eligibility. Following this, 243 potential articles were identified; however, 41 were excluded as they were either duplicates (12) or the full text was not written in English or the paper had no full text (29), leaving 202 SRs in this study. Figure 1 depicts the flow of SR selection.

### Characteristics of the 202 systematic reviews

Table 1 summarizes the characteristics of the 202 SRs, the full list for which is in the Additional file 2: Table S2. Among the SRs, 59 (29.2%), 53 (26.2%), 36 (17.8%), and 54 (26.7%) were published in the first, second, third, and fourth quarters of 2014, respectively. These SRs were published in 71 nursing journals, and the median number of SRs published in each journal was 2 (IQR: 1–3). More than 10 SRs were published in four journals in 2014, i.e., *International Journal of Nursing Studies*, *Journal of Clinical Nursing*, *Journal of Advanced Nursing*, and *Worldviews of Evidence-Based Nursing*. The scopes of the SRs were classified into four groups, i.e. Clinical Research (*n* = 145, 71.8%), Health Systems and Outcomes Research (*n* = 30, 14.9%), Nursing Education Research (*n* = 20, 9.9%), and Others (*n* = 7, 3.5%). A total of 172 (85.1%), 72 (35.6%), 153 (75.7%) and 149 (73.8%) of the SRs provided, respectively, dates of the last search, submission, acceptance and online publication. In addition, 198 (98.0%) of the SRs stated the databases used for the search, 37 (18.3%) included meta-analyses, and 108 (53.5%) disclosed the funding information.

**Table 1:** Characteristics of the 202 included systematic reviews

Factor	N (%)
Publication date	
• January to March	59 (29.2%)
• April to June	53 (26.2%)
• July to September	36 (17.8%)
• October to December	54 (26.7%)
• Number of pages in each SR	Median = 11 (IQR: 9–14)
Scope of the reviews	
• Clinical Research	145 (71.8%)
• Health Systems and Outcomes Research	30 (14.9%)
• Nursing Education Research	20 (9.9%)
• Others	7 (3.5%)
SR per journals (71 journals)	Median = 2(IQR: 1–3)
Stated the searched date in the SR	
• Yes	172 (85.1%)
• No	30 (14.9%)
Stated the databases used in the SR	
• Yes	198 (98.0%)
• No	4 (2.0%)
Number of records identified in database search	Median = 813 (IQR: 320.5–1789.5)
Number of articles included in the review	Median = 15 (IQR: 10–23)
Number of article in each review per year^	Median = 1.08 (IQR: 0.67–1.78)
Included meta-analysis in the SR	
• Yes	37 (18.3%)
• No	165 (81.7%)
Funding received^#^	
• Yes (Institutional/ Public)	55 (27.2%)
• Yes (Commercial)	5 (2.5%)
• No	48 (23.8%)
• Not reported	94 (46.5%)

^Dividing the total number of articles by the publication years between first and last published article

^#^PhD scholarship or studentship was not considered as funding

We attempted to contact the corresponding authors by e-mails for additional information but no e-mail addresses were provided in 13 papers. Therefore, a total of 189 e-mails were sent but 30 e-mail addresses were no longer valid. Among the 159 valid e-mail addresses, we received replies from 62 authors (response rate ~39.0%). Of them, 47 (75.8%) clarified that the journals were their first attempts while 10 (16.1%) clarified that they were their second attempts.

The methodological quality measured by the AMSTAR is summarized in Table 2. The median number of “Yes” obtained by the SRs was 3 (IQR: 2–5). A total of 139 (68.8%) SRs were classified as poor quality. For individual items, the percentage of “Yes” ranged from 0.0% (item 11) to 83.2% (item 6).

**Table 2:** Methodological quality of the 202 included systematic reviews accessed by AMSTAR

Item	Number of “Yes” (%)
1. Was an ‘a priori’ design provided?	6 (3.0%)
2. Was there duplicate study selection and data extraction?	55 (27.2%)
3. Was a comprehensive literature search performed?	140 (69.3%)
4. Was the status of publication (i.e. grey literature) used as an inclusion criterion?	43 (21.3%)
5. Was a list of studies (included and excluded) provided?	15 (7.4%)
6. Were the characteristics of the included studies provided?	168 (83.2%)
7. Was the scientific quality of the included studies assessed and documented?	103 (51.0%)
8. Was the scientific quality of the included studies used appropriately in formulating conclusions?	45 (22.3%)
9. Were the methods used to combine the findings of studies appropriate?	104 (51.5%)
10. Was the likelihood of publication bias assessed?	16 (7.9%)
11. Was the conflict of interest included?	0 (0.0%)
Overall	
Median of the number of “Yes” per study (Inter- quartile range)	3 (2–5)
Percentage of Poor Quality SR (less than 5 “Yes”)	139 (68.8%)
Percentage of Moderate Quality SR (5 to 8 “Yes”)	61 (30.2%)
Percentage of High Quality SR (9 to 11 “Yes”)	2 (1.0%)

The median (IQR) numbers of days taken from the last search to acceptance, to online publication, and to full publication of the SRs are 393 (212–609), 455 (273–654.5), and 669 (IQR: 427–915), respectively (Table 3). Secondly, the median (IQR) numbers of days from submission to acceptance, to online publication and to full publication were 153 (92–212), 212 (151–243) and 365 (243–486), respectively. However, it is noteworthy that only 72 SRs, around one-third of all included, provided their submission dates, thereby possibly rendering the results inaccurate. Lastly, the median (IQR) duration from acceptance to online publication was 59 (30–92) days, while that from online publication to full publication was 242 (153–365) days.

**Table 3:** Time taken from the search end-date to different publication status

Status	Mean (SD)	Median (1st – 3rd quartile)
Last searched to Submission (*n* = 52)	274.1 (289.6)	167.5 (53.5–427.0)
Last searched to Acceptance date (*n* = 135)	428.3 (283.1)	393 (212–609)
Last searched to Online Publication date (*n* = 129)	505.2 (302.9)	455 (273–654.5)
Last searched to Full Publication date (*n* = 171)	703.0 (321.4)	669 (427–915)
Submission to Acceptance date (*n* = 72)	157.9 (70.3)	153 (92–212)
Submission to Online Publication date (*n* = 58)	213.4 (110.6)	212 (151–243)
Submission to Full Publication date (n = 72)	388 (188.6)	365 (243–486)
Acceptance to Online Publication date (*n* = 104)	71.4 (81.7)	59 (30–92)
Acceptance to Full Publication date (*n* = 153)	271 (156.6)	242 (153–365)
Online to Full Publication date (*n* = 146)	213.2 (124.8)	212 (122.5–289)

No significant difference was observed in the time from last search to acceptance and to full publication (Table 4), with respect to factors such as the availability of the funding, holiday period submission, number of records identified in databases, and quality of studies.

**Table 4:** Comparison of median time from last search to acceptance or full publication for different variables

	Acceptance	Full publication
Funding^		
• Yes	153 (92–212)	334.5 (242.5–426)
• No	151 (91–214) U = 211, *p* = 0.852	396 (243–456.5) U = 182, *p* = 0.560
Holiday period submission		
• Yes (Apr, Jun- Aug, Dec)	153 (92–205)	335.5 (222–495)
• No	152 (92–213.5) U = 691, *p* = 0.484	365 (245–486.5) U = 694.5, *p* = 0.461
Number of records identified in database search		
• ≤1000	151 (92–184)	303 (242–487)
• >1000	212 (99–214) U = 687, *p* = 0.110	396 (303.75–456.75) U = 634.5, *p* = 0.349
Inclusion of meta-analysis		
• Yes	120 (91–214)	396 (245–518)
• No	153 (92–212) U = 448.5, *p* = 0.480	365 (243–471) U = 565, *p* = 0.432
Quality of the study		
• Poor	151 (92–212)	320 (242–516.5)
• Moderate or Good	182.5 (92–214) U = 453.5, *p* = 0.401	380.5 (288.5–485.5) U = 458, *p* = 0.435

^Some SRs did not disclose their funding information (see Table 1)

## Discussion

The identification of comprehensive and up-to-date SRs is important for researchers when evaluating evidence relevant to their topic of interest [25]. Our results revealed that the median time taken from the last search date to the online and full publication for the SRs in nursing journals were 455 and 669 days. These duration are markedly longer than those in medical journals (i.e., an average of 5.1 months from last search to first publication) [5]. The lack of timeliness in this context may jeopardize end-users such as guideline producers or policy makers in developing guidelines or revising policies as up-to-dated information may be missed.

In our study, the median time from the last search date to publication was 669 days (i.e., approximately 22.3 months). It has been advocated that systematic reviewers should update their literature search biennially to determine new studies for inclusion into a previously completed SR for updating of evidence [26]. Therefore, the time taken for publishing SRs in nursing journals is considered excessively long. Such time lags can actually be classified into three categories: from the last search date to submission, from submission to acceptance, and from acceptance to online publication.

The median time from the last search date to acceptance in nursing journals was 393 days, approximately 2.5-fold longer than those in Core Clinical Journals (5.1 months or approximately 153 days) [5]. The time taken can be classified into two periods: from the last search date to submission and from submission to acceptance. From our results, the median time from the last search date to submission was 167.5 days. The time taken was expended on the authors’ preparation of the manuscript and attempts of submission to journals. According to Polit and Beck [27], in conducting a SR, five steps are warranted after the literature search, i.e. selecting the studies, evaluating study quality, extracting and encoding data, analyzing and interpreting data (i.e., meta-analysis or synthesis), and writing the review. The time taken to complete these steps varies as this may depend on the researchers’ experiences. Therefore, having an experienced systematic reviewer within the authoring team may expedite these steps. For example, to register a new review with Cochrane Collaboration, a requirement is that at least one author must possess the experience in completing at least one Cochrane Review [28]. This requirement ensures the timeliness and quality of the review process.

In addition, authors are suggested to conduct preliminary selection of appropriate journals, from which the resultant review will more likely be accepted. This will avoid delays caused by multiple attempts when submitting to different journals [29, 30]. We contacted the corresponding authors of the 202 reviews and received replies from 62 authors, of whom 47 (75.8%) clarified that their papers were published in the journal in their first attempt. Therefore, such an effect should not be a critical factor among the SRs. However, as the SRs should be submitted before 2014, recall bias from the authors could not be ruled out.

The median time taken for SRs from submission to acceptance was found to be 153 days in this study. It includes the time for review and revision of the submitted manuscript. In comparison, the average time from submission to acceptance for journals from different disciplines was around 6.41 months (~192 days), which is longer than our result [31]. Some common problems identified for the delays in the review process include discrepancies with journal guidelines, submission during peak periods, reviewers’ delays, and failure to address reviewers’ comments or not submitting revisions on time [11]. For each of these problems, solutions are suggested to avoid unnecessary delays. As regards discrepancies with journal guidelines, although most authors adhere to the guidelines of the journals, when their manuscript is rejected by one journal, they may directly send the rejected manuscript to another without making any necessary changes. Further delays may follow when the editorial office of the second journal realizes stylistic and guideline breaches of the unedited manuscript and, in rejection, return it to the authors [32].

Some suggested that manuscript submission during holiday periods would probably lengthen the review process. There appears to be a propensity among some researchers to make use of the holiday to finalize and submit their manuscripts. Nevertheless, during this period, journal editors will find it difficult in recruiting potential reviewers, as some may be on vacation. [11]. However, no such observation can be concluded from our findings.

Reviewers’ delays may be resolved by shortening the timeframe for review. According to a recent study, some journals do not provide any deadline for their reviewers, whereas others allow up to 1 year [33]. In the same study, an experiment was conducted where all the participating journals were requested to shorten their current imposed review period by a minimum of 1 week. The results revealed a reduction in the editing time by at least 1 week on average, with several participating journals improving by more than 2 months [33]. In addition, the study revealed that reviewers were more likely to accept invitations with a shorter response timeframe [33].

The last problem concerns authors’ failures to address reviewers’ comments or not submitting revisions on time. The time estimated for nursing journals to complete the review is at least 8 weeks, when the editor will then make the first decision to accept or reject the manuscript, or to request revisions [10]. After receiving comments from the editor and reviewers, the authors should adequately address their comments by highlighting any changes in the manuscript and drafting a point-to-point response letter to expedite decision [10]. In addition, the authors should adhere to the deadline set by the journals to avoid their manuscripts being treated as new submissions or facing new reviewers [11]. The aforementioned strategies may collectively mitigate the four potential problems underlying excessive delays and thereby shorten the time taken from the last search date to the acceptance date. From the perspective of the journal editors, they may consider emulating the practice of Cochrane Database of Systematic Reviews where an updated literature search is required if the initial search was conducted more than 6 months prior to the review of the manuscript, as has been adopted by some General Medical Journals such as *Annals of Internal Medicine*. This will lessen the time lag between the search and the SR publication. Unless such an updated search before acceptance is made compulsory by journals, it is probable that some authors will not prioritize it since their results and, by extension, the discussion and conclusion may change.

The median time taken from acceptance to online publication of SRs published in nursing journals was 59 days. Such a duration may admittedly not be considered long but this is in fact twice the median time taken compared with papers found on PUBMED 2014 (approximately 25 days) [34]. This timing varies among different publishers and with current technology the situation should improve. The median time taken from online publication to full publication was 212 days (approximately 7 months). One possible reason underlying the time lags is the overwhelming number of accepted articles that is not commensurate with the publishing space in the journals. Nevertheless, this issue may become less critical with the increasing availability of the online early views in many journals for readers. Our results show that 149 (73.8%) SRs indicated their online publication dates; hence, it may be concluded that at least 73.8% of the SRs were made available earlier than the full publication date. Our results also suggest that the average time taken from acceptance to publication is 242 days. This is considered an improvement compared with the circumstance 10 years ago when the publication of SRs would usually take more than a year in most nursing journals [35].

According to Item 7 in the Preferred Reporting Items for Systematic Reviews and Meta-analyses (PRISMA) guidelines, authors of SRs should include all information sources (e.g., databases with dates of coverage, and the search and last search dates) [36]. Among the 202 SRs identified, 172 (85.1%) provided the last search date, which is slightly lower than Core Clinical Journals (90.3%) [5]. An overwhelming majority numbering 198 (98.0%) of the identified SRs stated the databases used for their search. This is comparable with the figure previously reported (98.3%) by Beller et al. [5]. Against this background, Liberati et al. [37] further suggested that this information would allow researchers to assess the scientific value of the review as the publication time lags may necessitate updates of SRs by the respective authors.

The quality of our included SRs was in general low with a median score of 3 out of 11. It is lower than the journals in Orthodontic (with mean scores 3.5 to 5.66) [23] or Pain related SRs (median: 6; IQR: 4–7) [13]. Two potential reasons underlie this low quality, the first of which concerns the lack of adherence among the authors to the PRISMA guideline in their reviews. Only 30 out of the 107 journals mandated the use of the PRISMA guideline [20]; accordingly, there might have been inadvertent omission of information in reporting their results. For example, the independent selection of papers might have been performed but this might not be reflected in the published review. The other potential reason concerns our stringent conformance to the AMSTAR criteria in examining the methodological quality. The generally low quality of our included SRs might be attributed to some AMSTAR criteria that were unfeasibly difficult to fulfill: for example, the last item on the AMSTAR checklist – “*Was the conflict of interest included?*” – requests the authors to indicate all sources of funding or support for the SRs and each of the studies included therein. The compliance to this item was often low and has indeed been reported to be approximately only 10% in the field of pain research [13]. Some studies would remove this item in evaluating the quality of SRs [38] whereas others would (more leniently) consider the disclosure of funding only for the authors but not for the individual studies included in the SRs [39]. Such refinements of definitions of the AMSTAR criteria may lead to a higher quality for the included studies.

### Strength and limitations

To the best of our knowledge, this is the first study focusing on the publication time lags of SRs published in nursing journals. The study sample is reasonably large with 202 SRs. Robust procedures have been adopted such as independent data extraction and evaluation, to ensure the accuracy of the results. Nevertheless, certain limitations of this study deserve attention. Firstly, only SRs published in 2014 were included as this study was conducted primarily in 2015 when 2014 was then the latest available full year. We did not include any preceding years because of limited resources but we believe that the observed pattern would be similar for 2015 or 2016. Secondly, the methodological quality of the SRs was generally low. Thirdly, non-English SRs were excluded due to language barriers of the authors; however, the time taken is expected to be similar or even longer. Fourthly, as a database search was used to identify the SRs, the possibility cannot be discounted that some articles might be missed if they had not included any words related to SR or mete-analysis. However, this method is considered acceptable. Fifthly, although the authors were contacted for additional information, only 2 questions were posted by e-mail to ensure an acceptable response rate. Further studies are recommended to explore the topic from both the journal editors’ and authors’ perspectives. Finally, given the absence of submission dates of most SRs, further analysis could not be conducted for most of them.

## Conclusion

Our study revealed a median difference of 669 days (approximately 22.3 months) between the last search date and the full publication date. With this information, journals should consider requesting authors of SRs to update their literature search before the acceptance of the SRs. SR users should also ascertain the time lag between the last search date of the reviews to ensure that the evidence is up-to-date for effective clinical decision-making.

## Additional files

Additional file 1: Table S1. Full list of journals indexed in Journal Citation Reports (Science Edition). (DOCX 24 kb)

Additional file 2: Table S2. Full list of included articles in this study. (DOCX 41 kb)
