# Supplementary material for: Is the information of systematic reviews published in nursing journals up-to-date? a cross-sectional study
Source: BMC Med Res Methodol. 2017 Nov 25;17:151. doi: 10.1186/s12874-017-0432-3 (PMC5702238; doi:10.1186/s12874-017-0432-3)
Supplement: Supplementary file 1 — Full list of journals indexed in Journal Citation Reports (Science Edition). (DOCX 24 kb) [file 12874_2017_432_MOESM1_ESM.docx]

| **Number** | **Full Journal Title** (web address to the journal’s homepage) |
| --- | --- |
| 1 | AAOHN JOURNAL (http://aaohn.org/) |
| 2 | Acta Paulista de Enfermagem (www.scielo.br/ape) |
| 3 | ADVANCES IN NURSING SCIENCE (journals.lww.com/advancesinnursingscience/pages/default.aspx) |
| 4 | Advances in Skin & Wound Care (journals.lww.com/aswcjournal/Pages/aboutthejournal.aspx) |
| 5 | AMERICAN JOURNAL OF CRITICAL CARE (ajcc.aacnjournals.org/) |
| 6 | AMERICAN JOURNAL OF NURSING (journals.lww.com/ajnonline/Pages/aboutthejournal.aspx) |
| 7 | APPLIED NURSING RESEARCH (https://www.journals.elsevier.com/applied-nursing-research) |
| 8 | Aquichan (www.scielo.org.co/scielo.php?script=sci_serial&pid=1657-5997) |
| 9 | ARCHIVES OF PSYCHIATRIC NURSING (www.psychiatricnursing.org/) |
| 10 | Asian Nursing Research (www.asian-nursingresearch.com/) |
| 11 | Assistenza Infermieristica e Ricerca (www.air-online.it/) |
| 12 | Australian Critical Care (www.australiancriticalcare.com/) |
| 13 | Australian Journal of Advanced Nursing (www.ajan.com.au/) |
| 14 | Australian Journal of Rural Health (ruralhealth.org.au/ajrh) |
| 15 | Bariatric Nursing and Surgical Patient Care (http://www.liebertpub.com/overview/bariatric-surgical-practice-and-patient-care/172/) |
| 16 | Biological Research for Nursing (journals.sagepub.com/home/brn) |
| 17 | BIRTH-ISSUES IN PERINATAL CARE (onlinelibrary.wiley.com/journal/10.1111/(ISSN)1523-536X) |
| 18 | CANCER NURSING (http://journals.lww.com/cancernursingonline/pages/default.aspx?desktopMode=true) |
| 19 | CIN-COMPUTERS INFORMATICS NURSING (journals.lww.com/cinjournal/pages/default.aspx) |
| 20 | Clinical Journal of Oncology Nursing (https://cjon.ons.org/content/about-cjon) |
| 21 | Clinical Nurse Specialist (http://journals.lww.com/cns-journal/pages/default.aspx) |
| 22 | Clinical Nursing Research (journals.sagepub.com/home/cnr) |
| 23 | Collegian (www.collegianjournal.com/) |
| 24 | Contemporary Nurse (www.tandfonline.com/loi/rcnj20) |
| 25 | Critical Care Nurse (ccn.aacnjournals.org/) |
| 26 | Critical Care Nursing Clinics of North America (www.ccnursing.theclinics.com/) |
| 27 | EUROPEAN JOURNAL OF CANCER CARE (onlinelibrary.wiley.com/journal/10.1111/(ISSN)1365-2354) |
| 28 | European Journal of Cardiovascular Nursing (journals.sagepub.com/home/cnu) |
| 29 | European Journal of Oncology Nursing (www.ejoncologynursing.com/) |
| 30 | Gastroenterology Nursing (journals.lww.com/gastroenterologynursing/pages/default.aspx) |
| 31 | GERIATRIC NURSING (www.gnjournal.com/) |
| 32 | HEART & LUNG (www.heartandlung.org/) |
| 33 | Holistic Nursing Practice (journals.lww.com/hnpjournal/pages/default.aspx ) |
| 34 | International Emergency Nursing (https://www.journals.elsevier.com/international-emergency-nursing) |
| 35 | International Journal of Mental Health Nursing (onlinelibrary.wiley.com/journal/10.1111/(ISSN)1447-0349) |
| 36 | International Journal of Nursing Knowledge (onlinelibrary.wiley.com/journal/10.1111/(ISSN)2047-3095) |
| 37 | International Journal of Nursing Practice (onlinelibrary.wiley.com/journal/10.1111/(ISSN)1440-172X) |
| 38 | INTERNATIONAL JOURNAL OF NURSING STUDIES (www.journalofnursingstudies.com/) |
| 39 | International Journal of Nursing Terminologies and Classifications (onlinelibrary.wiley.com/journal/10.1111/(ISSN)2047-3095/issues) |
| 40 | INTERNATIONAL NURSING REVIEW (onlinelibrary.wiley.com/journal/10.1111/%28ISSN%291466-7657) |
| 41 | JANAC-JOURNAL OF THE ASSOCIATION OF NURSES IN AIDS CARE (www.nursesinaidscarejournal.org/) |
| 42 | Japan Journal of Nursing Science (onlinelibrary.wiley.com/journal/10.1111/(ISSN)1742-7924) |
| 43 | JOGNN-JOURNAL OF OBSTETRIC GYNECOLOGIC AND NEONATAL NURSING (www.jognn.org/) |
| 44 | Journal for Specialists in Pediatric Nursing (onlinelibrary.wiley.com/journal/10.1111/(ISSN)1744-6155) |
| 45 | Journal of Addictions Nursing (journals.lww.com/jan/pages/default.aspx) |
| 46 | JOURNAL OF ADVANCED NURSING (onlinelibrary.wiley.com/journal/10.1111/(ISSN)1365-2648) |
| 47 | Journal of Cardiovascular Nursing (journals.lww.com/jcnjournal/pages/default.aspx) |
| 48 | Journal of Child Health Care (journals.sagepub.com/home/chc) |
| 49 | JOURNAL OF CLINICAL NURSING (onlinelibrary.wiley.com/journal/10.1111/(ISSN)1365-2702) |
| 50 | Journal of Community Health Nursing (www.tandfonline.com/loi/hchn20) |
| 51 | JOURNAL OF CONTINUING EDUCATION IN NURSING (https://www.healio.com/nursing/journals/jcen) |
| 52 | Journal of Emergency Nursing (www.jenonline.org/) |
| 53 | Journal of Family Nursing (journals.sagepub.com/home/jfn) |
| 54 | Journal of Gerontological Nursing (https://geriatricscareonline.org/ProductAbstract/Journal-of-Gerontological-Nursing/J003) |
| 55 | Journal of Hospice & Palliative Nursing (journals.lww.com/jhpn/Pages/aboutthejournal.aspx) |
| 56 | JOURNAL OF HUMAN LACTATION (journals.sagepub.com/home/jhl) |
| 57 | Journal of Korean Academy of Nursing (https://koreamed.org/JournalVolume.php?id=6) |
| 58 | JOURNAL OF MIDWIFERY & WOMENS HEALTH (onlinelibrary.wiley.com/journal/10.1111/%28ISSN%291542-2011) |
| 59 | JOURNAL OF NEUROSCIENCE NURSING (journals.lww.com/jnnonline/Pages/default.aspx?PAPNotFound=true) |
| 60 | JOURNAL OF NURSING ADMINISTRATION (journals.lww.com/jonajournal/pages/default.aspx) |
| 61 | JOURNAL OF NURSING CARE QUALITY (journals.lww.com/jncqjournal/pages/default.aspx) |
| 62 | JOURNAL OF NURSING EDUCATION (https://www.healio.com/nursing/journals/jne) |
| 63 | Journal of Nursing Management (onlinelibrary.wiley.com/journal/10.1111/(ISSN)1365-2834) |
| 64 | Journal of Nursing Research (journals.lww.com/jnr-twna/pages/default.aspx) |
| 65 | JOURNAL OF NURSING SCHOLARSHIP (onlinelibrary.wiley.com/journal/10.1111/(ISSN)1547-5069) |
| 66 | Journal of Pediatric Health Care (www.jpedhc.org/) |
| 67 | Journal of Pediatric Nursing-Nursing Care of Children & Families (www.pediatricnursing.org/) |
| 68 | Journal of Pediatric Oncology Nursing (journals.sagepub.com/home/jpo) |
| 69 | Journal of PeriAnesthesia Nursing (www.jopan.org/) |
| 70 | JOURNAL OF PERINATAL & NEONATAL NURSING (journals.lww.com/jpnnjournal/pages/default.aspx) |
| 71 | JOURNAL OF PROFESSIONAL NURSING (www.professionalnursing.org/) |
| 72 | Journal of Psychiatric and Mental Health Nursing (onlinelibrary.wiley.com/journal/10.1111/(ISSN)1365-2850) |
| 73 | JOURNAL OF PSYCHOSOCIAL NURSING AND MENTAL HEALTH SERVICES (https://www.healio.com/psychiatry/journals/jpn) |
| 74 | Journal of School Nursing (journals.sagepub.com/home/jsn) |
| 75 | Journal of the American Academy of Nurse Practitioners (onlinelibrary.wiley.com/journal/10.1002/(ISSN)2327-6924/issues) |
| 76 | Journal of Tissue Viability (www.journaloftissueviability.com/) |
| 77 | Journal of Transcultural Nursing (journals.sagepub.com/home/tcn) |
| 78 | Journal of Wound Ostomy and Continence Nursing (journals.lww.com/jwocnonline/pages/default.aspx) |
| 79 | MCN-The American Journal of Maternal-Child Nursing (journals.lww.com/mcnjournal/pages/default.aspx) |
| 80 | MIDWIFERY (https://www.journals.elsevier.com/midwifery) |
| 81 | Nephrology Nursing Journal (https://www.annanurse.org/resources/products/nephrology-nursing-journal) |
| 82 | NURSE EDUCATION TODAY (www.nurseeducationtoday.com/) |
| 83 | Nurse Educator (journals.lww.com/nurseeducatoronline/pages/default.aspx) |
| 84 | Nursing & Health Sciences (onlinelibrary.wiley.com/journal/10.1111/(ISSN)1442-2018) |
| 85 | NURSING CLINICS OF NORTH AMERICA (www.nursing.theclinics.com/) |
| 86 | NURSING ETHICS (journals.sagepub.com/home/nej) |
| 87 | Nursing in Critical Care (onlinelibrary.wiley.com/journal/10.1111/(ISSN)1478-5153) |
| 88 | Nursing Inquiry (onlinelibrary.wiley.com/journal/10.1111/(ISSN)1440-1800) |
| 89 | NURSING OUTLOOK (www.nursingoutlook.org/) |
| 90 | Nursing Philosophy (http://onlinelibrary.wiley.com/journal/10.1111/(ISSN)1466-769X) |
| 91 | NURSING RESEARCH (journals.lww.com/nursingresearchonline/pages/default.aspx) |
| 92 | NURSING SCIENCE QUARTERLY (journals.sagepub.com/home/nsq) |
| 93 | ONCOLOGY NURSING FORUM (https://onf.ons.org/) |
| 94 | Orthopaedic Nursing (journals.lww.com/orthopaedicnursing/pages/default.aspx) |
| 95 | Pain Management Nursing (www.painmanagementnursing.org/) |
| 96 | PERSPECTIVES IN PSYCHIATRIC CARE (onlinelibrary.wiley.com/journal/10.1111/(ISSN)1744-6163) |
| 97 | Pflege (https://www.editorialmanager.com/pflege/default.aspx) |
| 98 | PUBLIC HEALTH NURSING (http://onlinelibrary.wiley.com/journal/10.1111/(ISSN)1525-1446) |
| 99 | Rehabilitation Nursing (http://onlinelibrary.wiley.com/journal/10.1002/(ISSN)2048-7940) |
| 100 | Research and Theory for Nursing Practice (www.springerpub.com/research-and-theory-for-nursing-practice.html) |
| 101 | Research in Gerontological Nursing (https://www.healio.com/nursing/journals/rgn) |
| 102 | RESEARCH IN NURSING & HEALTH (http://onlinelibrary.wiley.com/journal/10.1002/(ISSN)1098-240X) |
| 103 | Revista da Escola de Enfermagem da USP (www.scielo.br/reeusp) |
| 104 | WESTERN JOURNAL OF NURSING RESEARCH (journals.sagepub.com/home/wjn) |
| 105 | Women and Birth (https://www.journals.elsevier.com/women-and-birth/) |
| 106 | Workplace Health & Safety (journals.sagepub.com/home/whs) |
| 107 | Worldviews on Evidence-Based Nursing (onlinelibrary.wiley.com/journal/10.1111/(ISSN)1741-6787) |
