# Supplementary material for: Is the information of systematic reviews published in nursing journals up-to-date? a cross-sectional study
Source: BMC Med Res Methodol. 2017 Nov 25;17:151. doi: 10.1186/s12874-017-0432-3 (PMC5702238; doi:10.1186/s12874-017-0432-3)
Supplement: Supplementary file 2 — Full list of included articles in this study. (DOCX 41 kb) [file 12874_2017_432_MOESM2_ESM.docx]

Supplementary Table S1

1. S. S. McAlvin and A. Carew-Lyons Family presence during resuscitation and invasive procedures in pediatric critical care: a systematic review American Journal of Critical Care 2014 23 477-484
2. X. Yin Y. Zhao and X. Zhu Comparison of fast track protocol and standard care in patients undergoing elective open colorectal resection: a meta-analysis update Applied Nursing Research 2014 27 e20-6
3. P. Seong-Hi H. Kuem Sun and K. Chang-Bum Effects of exercise programs on depressive symptoms quality of life and self-esteem in older people: A systematic review of randomized controlled trials Applied Nursing Research 2014 27 219-226
4. K. Phillips K. Keane and B. E. Wolfe Peripheral Brain Derived Neurotrophic Factor (BDNF) in Bulimia Nervosa: A Systematic Review Archives of Psychiatric Nursing 2014 28 108-113
5. S. J. Chang S. Choi S. A. Kim and M. Song Intervention Strategies Based on Information-Motivation-Behavioral Skills Model for Health Behavior Change: A Systematic Review Asian Nursing Research 2014 8 172-181
6. S. O'Connor and S. Murphy Chronic Venous Leg Ulcers: Is Topical Zinc the Answer? A Review of the Literature Advances in Skin & Wound Care 2014 27 35-44
7. S. H. Park K. S. Han and C. B. Kang Relaxation Therapy for Irritable Bowel Syndrome: A Systematic Review Asian Nursing Research 2014 8 182-192
8. K. M. Murdoch B. Mitra S. Lambert and B. Erbas What is the seasonal distribution of community acquired pneumonia over time? A systematic review Australasian Emergency Nursing Journal 2014 17 30-42
9. Banbury A. Roots and S. Nancarrow Rapid review of applications of e-health and remote monitoring for rural residents Australian Journal of Rural Health 2014 22 211-222
10. S. Ruggiero and N. S. Redeker Effects of Napping on Sleepiness and Sleep-Related Performance Deficits in Night-Shift Workers A Systematic Review Biological Research for Nursing 2014 16 134-142
11. M. Rossignol N. Chaillet F. Boughrassa and J. M. Moutquin Interrelations Between Four Antepartum Obstetric Interventions and Cesarean Delivery in Women at Low Risk: A Systematic Review and Modeling of the Cascade of Interventions Birth-Issues in Perinatal Care 2014 41 70-78
12. E. Neville M. C. McKinley V. A. Holmes D. Spence and J. V. Woodside The Effectiveness of Weight Management Interventions in Breastfeeding Women-A Systematic Review and Critical Evaluation Birth-Issues in Perinatal Care 2014 41 223-236
13. S. L. Robb and D. Hanson-Abromeit A Review of Supportive Care Interventions to Manage Distress in Young Children With Cancer and Parents Cancer Nursing 2014 37 E1-E26
14. P. J. Gilbar Intrathecal Chemotherapy Potential for Medication Error Cancer Nursing 2014 37 299-309
15. L. Todd M. C. Moskowitz A. Ottati and M. Feuerstein Stressors Stress Response and Cancer Recurrence A Systematic Review Cancer Nursing 2014 37 114-125
16. M. R. Fu J. Deng and J. M. Armer Putting Evidence Into Practice: Cancer-Related Lymphedema Clinical Journal of Oncology Nursing 2014 18 68-79
17. Finfgeld-Connett Intimate Partner Abuse Among Older Women: Qualitative Systematic Review Clinical Nursing Research 2014 23 664-683
18. M. Engwall I. Fridh I. Bergbom and B. Lindahl Let There Be Light And Darkness Critical Care Nursing Quarterly 2014 37 273-298
19. H. Cramer R. Lauche P. Klose G. Dobos and J. Langhorst A systematic review and meta-analysis of exercise interventions for colorectal cancer patients European Journal of Cancer Care 2014 23 Mar-14
20. C. Handberg C. V. Nielsen and K. Lomborg Men's reflections on participating in cancer rehabilitation: a systematic review of qualitative studies 2000-2013 European Journal of Cancer Care 2014 23 159-172
21. S. Kenderian E. K. Stephens and A. Jatoi Ostomies in rectal cancer patients: what is their psychosocial impact? European Journal of Cancer Care 2014 23 328-332
22. K. Damm A. Vogel and A. Prenzler Preferences of colorectal cancer patients for treatment and decision-making: a systematic literature review European Journal of Cancer Care 2014 23 762-772
23. M. H. Mackay P. A. Ratner M. Nguyen M. Percy P. Galdas and G. Grunau Inconsistent measurement of acute coronary syndrome patients' pre-hospital delay in research: A review of the literature European Journal of Cardiovascular Nursing 2014 13 483-493
24. Conway V. Schadewaldt R. Clark C. Ski D. R. Thompson K. Kynoch and L. Doering The effectiveness of non-pharmacological interventions in improving psychological outcomes for heart transplant recipients: A systematic review European Journal of Cardiovascular Nursing 2014 13 108-115
25. Q. P. Li and A. Y. Loke A literature review on the mutual impact of the spousal caregiver-cancer patients dyads: 'Communication' 'reciprocal influence' and 'caregiver-patient congruence' European Journal of Oncology Nursing 2014 18 58-65
26. L. Deckx M. van den Akker and F. Buntinx Risk factors for loneliness in patients with cancer: A systematic literature review and meta-analysis European Journal of Oncology Nursing 2014 18 466-477
27. J. Purath A. Keck and C. E. Fitzgerald Motivational interviewing for older adults in primary care: A systematic review Geriatric Nursing 2014 35 219-224
28. S. S. Al-Zaiti J. A. Fallavollita Y.-W. B. Wu M. R. Tomita and M. G. Carey Electrocardiogram-based predictors of clinical outcomes: A meta-analysis of the prognostic value of ventricular repolarization Heart & Lung 2014 43 516-526
29. H. Yu C. Lin H. Fan and Z. Li The efficacy of noninvasive ventilation in managing postextubation respiratory failure: A meta-analysis Heart & Lung 2014 43 99-104
30. N. M. Albert Use of novel oral anticoagulants for patients with atrial fibrillation: Systematic review and clinical implications Heart & Lung 2014 43 48-59
31. Happell L. Byrne M. McAllister D. Lampshire C. Roper C. J. Gaskin G. Martin D. Wynaden B. McKenna R. Lakeman C. Platania-Phung and H. Hamer Consumer involvement in the tertiary-level education of mental health professionals: A systematic review International Journal of Mental Health Nursing 2014 23 Mar-16
32. Hegedus and B. Kozel Does adherence therapy improve medication adherence among patients with schizophrenia? A systematic review International Journal of Mental Health Nursing 2014 23 490-497
33. R. Stanton and B. Happell Exercise for mental illness: A systematic review of inpatient studies International Journal of Mental Health Nursing 2014 23 232-242
34. Walsh E. McCann S. Gilbody and E. Hughes Promoting HIV and sexual safety behaviour in people with severe mental illness: A systematic review of behavioural interventions International Journal of Mental Health Nursing 2014 23 344-354
35. L. Mabey and G. van Servellen Treatment of post-traumatic stress disorder in patients with severe mental illness: A review International Journal of Mental Health Nursing 2014 23 42-50
36. S. F. Ali and M. P. Muhammad Patient Preference Regarding their Role in Clinical Decision Making Process: A Systematic Review International Journal of Nursing Education 2014 6 192-195
37. J. M. Li V. Drury and B. Taylor A systematic review of the experience of older women living and coping with type 2 diabetes International Journal of Nursing Practice 2014 20 126-134
38. N. Blay C. M. Duffield R. Gallagher and M. Roche A systematic review of time studies to assess the impact of patient transfers on nurse workload International Journal of Nursing Practice 2014 20 662-673
39. Larkin V. Lopez and E. Aromataris Managing cancer-related fatigue in men with prostate cancer: A systematic review of non-pharmacological interventions International Journal of Nursing Practice 2014 20 549-560
40. N. R. Parenti M. L./Iannone P./Percudani D./Dowding D. A systematic review on the validity and reliability of an emergency department triage scale the Manchester Triage System International Journal of Nursing Studies 2014 51 1062-9
41. T. L. S. S. Dorfman E./Rempel G. R./Scott S. D./Hartling L. An evaluation of instruments for scoring physiological and behavioral cues of pain non-pain related distress and adequacy of analgesia and sedation in pediatric mechanically ventilated patients: A systematic review International Journal of Nursing Studies 2014 51 654-76
42. R. M. Mohler G. Attitudes of nurses towards the use of physical restraints in geriatric care: a systematic review of qualitative and quantitative studies International Journal of Nursing Studies 2014 51 274-88
43. K. Westermann A./Harling M./Nienhaus A. Burnout intervention studies for inpatient elderly care nursing staff: systematic literature review International Journal of Nursing Studies 2014 51 63-71
44. W. Coolbrandt H./Aertgeerts B./Van der Elst E./Laenen A./Dierckx de Casterle B./van Achterberg T./Milisen K. Characteristics and effectiveness of complex nursing interventions aimed at reducing symptom burden in adult patients treated with chemotherapy: a systematic review of randomized controlled trials International Journal of Nursing Studies 2014 51 495-510
45. M. H. Mohammady K./Akbari Sari A./Zolfaghari M./Janani L. Early ambulation after diagnostic transfemoral catheterisation: a systematic review and meta-analysis International Journal of Nursing Studies 2014 51 39-50
46. S. B. W. Tan A. F./Kelly D. Effectiveness of multidisciplinary interventions to improve the quality of life for people with Parkinson's disease: a systematic review International Journal of Nursing Studies 2014 51 166-74
47. S. L. Tastan G. C./Keenan G. M./Stifter J./McKinney D./Fahey L./Lopez K. D./Yao Y./Wilkie D. J. Evidence for the existing American Nurses Association-recognized standardized nursing terminologies: a systematic review International Journal of Nursing Studies 2014 51 1160-70
48. K. S. Lowther L./Harding R./Higginson I. J. Experience of persistent psychological symptoms and perceived stigma among people with HIV on antiretroviral therapy (ART): a systematic review International Journal of Nursing Studies 2014 51 1171-89
49. W. Wilkinson L./Ritchie L. Factors influencing the ability to self-manage diabetes for adults living with type 1 or 2 diabetes International Journal of Nursing Studies 2014 51 111-22
50. S. Wassenaar J./Schoonhoven L. Factors promoting intensive care patients' perception of feeling safe: a systematic review International Journal of Nursing Studies 2014 51 261-73
51. S. Brownie L. Horstmanshof and R. Garbutt Factors that impact residents' transition and psychological adjustment to long-term aged care: A systematic literature review International Journal of Nursing Studies 2014 51 1654-1666
52. M. H. Lahti H./Valimaki M. Impact of e-learning on nurses' and student nurses knowledge skills and satisfaction: a systematic review and meta-analysis International Journal of Nursing Studies 2014 51 136-49
53. W. C. Liu J./Thomas S. A. Interventions on mealtime difficulties in older adults with dementia: a systematic review International Journal of Nursing Studies 2014 51 14-27
54. F. Murphy M./Prieto J. Interventions to minimise the initial use of indwelling urinary catheters in acute care: a systematic review International Journal of Nursing Studies 2014 51 41365
55. F. S. Wang Y. L./Zang H. X. Music therapy improves sleep quality in acute and chronic sleep disorders: a meta-analysis of 10 randomized studies International Journal of Nursing Studies 2014 51 51-62
56. R. D. Samuriwo D. Nurses' pressure ulcer related judgements and decisions in clinical practice: a systematic review International Journal of Nursing Studies 2014 51 1667-85
57. N. Gorecki J./Lamping D. L./Alavi Y./Brown J. M. Patient-reported outcome measures for chronic wounds with particular reference to pressure ulcer research: a systematic review International Journal of Nursing Studies 2014 51 157-65
58. R. G. V. K. Ettema H./Peelen L. M./Kalkman C. J./Schuurmans M. J. Preadmission interventions to prevent postoperative complications in older cardiac surgery patients: a systematic review International Journal of Nursing Studies 2014 51 251-60
59. Brugnolli E./Canzan F./Saiani L. Securing of naso-gastric tubes in adult patients: a review International Journal of Nursing Studies 2014 51 943-50
60. R. B. Harrison Y./Hall J./Bosanquet K./Harden M./Iedema R. The contribution of nurses to incident disclosure: a narrative review International Journal of Nursing Studies 2014 51 334-45
61. L. N. Q. Kong B./Zhou Y. Q./Mou S. Y./Gao H. M. The effectiveness of problem-based learning on development of nursing students' critical thinking: a systematic review and meta-analysis International Journal of Nursing Studies 2014 51 458-69
62. S. C. D. Gielen J./Francke A. L./Mistiaen P./Kroezen M. The effects of nurse prescribing: a systematic review International Journal of Nursing Studies 2014 51 1048-61
63. H. L. Chien K. L./Chien H. T./Liu H. E. The effects of psychosocial strategies on anxiety and depression of patients diagnosed with prostate cancer: a systematic review International Journal of Nursing Studies 2014 51 28-38
64. L. A. Daouk-Oyry A. L./Otaki F./Dumit N. Y./Osman I. The JOINT model of nurse absenteeism and turnover: a systematic review International Journal of Nursing Studies 2014 51 93-110
65. S. May A./Hunt K. The nursing work of hospital-based clinical practice guideline implementation: an explanatory systematic review using Normalisation Process Theory International Journal of Nursing Studies 2014 51 289-99
66. Jung S. Shin and H. Kim A fall prevention guideline for older adults living in long-term care facilities International Nursing Review 2014 61 525-533
67. J. Joseph D. Basu M. Dandapani and N. Krishnan Are nurse-conducted brief interventions (NCBIs) efficacious for hazardous or harmful alcohol use? A systematic review International Nursing Review 2014 61 203-210
68. G. Moscou-Jackson Y. Commodore-Mensah J. Farley and M. DiGiacomo Smoking-Cessation Interventions in People Living With HIV Infection: A Systematic Review Janac-Journal of the Association of Nurses in Aids Care 2014 25 32-45
69. Y. Yang State of the Science: The Efficacy of a Multicomponent Intervention for ART Adherence Among People Living With HIV Janac-Journal of the Association of Nurses in Aids Care 2014 25 297-308
70. M. R. Temple J. C. A systematic review of interventions to promote physical activity in the preschool setting Journal for Specialists in Pediatric Nursing 2014 19 274-84
71. S. B. Quelly Childhood obesity prevention: a review of school nurse perceptions and practices Journal for Specialists in Pediatric Nursing 2014 19 198-209
72. G. Balmer F./Dunn J. A qualitative systematic review exploring lay understanding of cancer by adults without a cancer diagnosis Journal of Advanced Nursing 2014 70 1688-701
73. L. G. H.-E. Park J./Dracup K. A quantitative systematic review of the efficacy of mobile phone interventions to improve medication adherence Journal of Advanced Nursing 2014 70 1932-53
74. Close M. Sinclair S. D. Liddle E. Madden J. E. M. McCullough and C. Hughes A systematic review investigating the effectiveness of Complementary and Alternative Medicine ( CAM) for the management of low back and/or pelvic pain ( LBPP) in pregnancy Journal of Advanced Nursing 2014 70 1702-1716
75. M. K. Hokka P./Polkki T. A systematic review: non-pharmacological interventions in treating pain in patients with advanced cancer Journal of Advanced Nursing 2014 70 1954-69
76. L. R. Flanagan B./Jack B./Shaw C./Williams K. S./Chung A./Barrett J. Factors with the management of incontinence and promotion of continence in older people in care homes Journal of Advanced Nursing 2014 70 476-96
77. Y. J. C. Chieng W. C./Klainin-Yobas P./He H. G. Perioperative anxiety and postoperative pain in children and adolescents undergoing elective surgical procedures: a quantitative systematic review Journal of Advanced Nursing 2014 70 243-55
78. T. M. H. Giles K. L. Qualitative systematic review: the unique experiences of the nurse-family member when a loved one is admitted with a critical illness Journal of Advanced Nursing 2014 70 1451-64
79. K. M. Missen L./Beauchamp A. Satisfaction of newly graduated nurses enrolled in transition-to-practice programmes in their first year of employment: a systematic review Journal of Advanced Nursing 2014 70 2419-33
80. K. T. Leung L./Waters D. Systematic review of instruments for measuring nurses' knowledge skills and attitudes for evidence-based practice Journal of Advanced Nursing 2014 70 2181-95
81. J. M.-S. Caro-Bautista F. J./Morales-Asencio J. M. Systematic review of the psychometric properties and theoretical grounding of instruments evaluating self-care in people with type 2 diabetes mellitus Journal of Advanced Nursing 2014 70 1209-27
82. M. K. Kangasniemi H./Pietila A. M. Towards environmentally responsible nursing: a critical interpretive synthesis Journal of Advanced Nursing 2014 70 1465-78
83. Walton-Moss L. Samuel T. H. Nguyen Y. Commodore-Mensah M. J. Hayat and S. L. Szanton Community-Based Cardiovascular Health Interventions in Vulnerable Populations A Systematic Review Journal of Cardiovascular Nursing 2014 29 293-307
84. T. L. Blair Device Diagnostics and Early Identification of Acute Decompensated Heart Failure A Systematic Review Journal of Cardiovascular Nursing 2014 29 68-81
85. J. K. Herr J. Salyer D. E. Lyon L. Goodloe C. Schubert and D. G. Clement Heart Failure Symptom Relationships A Systematic Review Journal of Cardiovascular Nursing 2014 29 416-422
86. H. R. Han H. J. Song T. Nguyen and M. T. Kim Measuring Self-care in Patients With Hypertension A Systematic Review of Literature Journal of Cardiovascular Nursing 2014 29 55-67
87. U. H. Timlin H./Heino R./Kyngas H. A systematic narrative review of the literature: adherence to pharmacological and nonpharmacological treatments among adolescents with mental disorders Journal of Clinical Nursing 2014 23 3321-34
88. P. Holly E. B. A systematic review on the transfer of information during nurse transitions in care Journal of Clinical Nursing 2014 23 2387-95
89. L. A. R. Hoga J. R./Sato P. M./Nunes M. C./Borges A. L. Adult men's beliefs values attitudes and experiences regarding contraceptives: a systematic review of qualitative studies Journal of Clinical Nursing 2014 23 927-39
90. M. A. Mohammady F./Sari A. A./Zolfaghari M. Bed rest duration after sheath removal following percutaneous coronary interventions: a systematic review and meta-analysis Journal of Clinical Nursing 2014 23 1476-85
91. H. Hofmann and S. Hahn Characteristics of nursing home residents and physical restraint: a systematic literature review Journal of Clinical Nursing 2014 23 3012-3024
92. H. Nelson M. B. Different context different results: venous ulcer healing and the use of two high-compression technologies Journal of Clinical Nursing 2014 23 768-73
93. P. V. Copanitsanou K. Effects of education of paediatric patients undergoing elective surgical procedures on their anxiety - a systematic review Journal of Clinical Nursing 2014 23 940-54
94. F. Wallstrom G. H. Facilitating early recovery of bowel motility after colorectal surgery: a systematic review Journal of Clinical Nursing 2014 23 24-44
95. N. A. D. Al Abed P. M./Hickman L. D. Healthcare needs of older Arab migrants: a systematic review Journal of Clinical Nursing 2014 23 1770-84
96. W. B. Zhang K. L./While A. E. Nurses' attitudes towards medical devices in healthcare delivery: a systematic review Journal of Clinical Nursing 2014 23 2725-39
97. J. Clarke Nursing practice in stroke rehabilitation: systematic review and meta-ethnography Journal of Clinical Nursing 2014 23 1201-26
98. Y. J. Kim Relationship of trauma centre characteristics and patient outcomes: a systematic review Journal of Clinical Nursing 2014 23 301-14
99. T.-T. Zhang S.-S. Tang and L.-J. Fu The effectiveness of different concentrations of chlorhexidine for prevention of ventilator-associated pneumonia: a meta-analysis Journal of Clinical Nursing 2014 23 1461-1475
100. Eidhammer F. A. J. Fluttert and S. Bjorkly User involvement in structured violence risk management within forensic mental health facilities - a systematic literature review Journal of Clinical Nursing 2014 23 2716-2724
101. P. S. Lin M. K. Viscardi and M. D. McHugh Factors Influencing Job Satisfaction of New Graduate Nurses Participating in Nurse Residency Programs: A Systematic Review Journal of Continuing Education in Nursing 2014 45 439-450
102. N. K. Gillis C. Arslanian-Engoren and L. M. Struble ACUTE CORONARY SYNDROMES IN OLDER ADULTS: A REVIEW OF LITERATURE Journal of Emergency Nursing 2014 40 270-275
103. U. Ostlund and C. Persson Examining Family Responses to Family Systems Nursing Interventions: An Integrative Review Journal of Family Nursing 2014 20 259-286
104. M. C. Lee K. A. Hinderer and K. A. Kehl A Systematic Review of Advance Directives and Advance Care Planning in Chinese People From Eastern and Western Cultures Journal of Hospice & Palliative Nursing 2014 16 75-85
105. R. Carton and J. E. Hupcey The Forgotten Mourners Addressing Health Care Provider Grief-A Systematic Review Journal of Hospice & Palliative Nursing 2014 16 291-303
106. R. Giglia and C. Binns The Effectiveness of the Internet in Improving Breastfeeding Outcomes: A Systematic Review Journal of Human Lactation 2014 30 156-160
107. M. D. Avery M. A. Saftner B. Larson and E. V. Weinfurter A Systematic Review of Maternal Confidence for Physiologic Birth: Characteristics of Prenatal Care and Confidence Measurement Journal of Midwifery & Womens Health 2014 59 586-595
108. Y. M. Chau S. West and V. Mapedzahama Night Work and the Reproductive Health of Women: An Integrated Literature Review Journal of Midwifery & Womens Health 2014 59 113-126
109. L.-P. Feng H.-L. Chen and M.-Y. Shen Breastfeeding and the Risk of Ovarian Cancer: A Meta-Analysis Journal of Midwifery & Women's Health 2014 59 428-437
110. K. Oikarinen M./Kyngas H. A framework of counseling for patients with stroke in nursing: a narrative literature review Journal of Neuroscience Nursing 2014 46 E3-E14
111. M. D. Mitchell J. G. Lavenberg R. L. Trotta and C. A. Umscheid Hourly Rounding to Improve Nursing Responsiveness A Systematic Review Journal of Nursing Administration 2014 44 462-472
112. D. Milbrath R. Linroth J. Wilhelmy and A. Pate A Method of Comparing Effectiveness of Mattresses for Pressure Management for Pediatric Patients Journal of Nursing Care Quality 2014 29 66-73
113. E. Franklin and C. S. Lee Effectiveness of simulation for improvement in self-efficacy among novice nurses: a meta-analysis Journal of Nursing Education 2014 53 607-14
114. L. Holm and E. Severinsson Effective nursing leadership of older persons in the community a systematic review Journal of Nursing Management 2014 22 211-224
115. S. Lartey G. Cummings and J. Profetto-McGrath Interventions that promote retention of experienced registered nurses in health care settings: a systematic review Journal of Nursing Management 2014 22 1027-1041
116. Aarthun and K. Akerjordet Parent participation in decision- making in health- care services for children: an integrative review Journal of Nursing Management 2014 22 177-191
117. M. Chen and M. F. Lou The effectiveness and application of mentorship programmes for recently registered nurses: a systematic review Journal of Nursing Management 2014 22 433-442
118. Cicolini D. Comparcini and V. Simonetti Workplace empowerment and nurses' job satisfaction: a systematic literature review Journal of Nursing Management 2014 22 855-871
119. Yueh-Juen and L. Feng-Yu Effectiveness of Propolis on Oral Health: A Meta-Analysis Journal of Nursing Research (Lippincott Williams & Wilkins) 2014 22 221-230
120. W. Yu-Ming S. Bih-Ching S. Fetzer and C. Ying-Ju Parenting Style of Women Who Conceived Using In Vitro Fertilization: A Meta-Analysis Journal of Nursing Research (Lippincott Williams & Wilkins) 2014 22 69-80
121. M. N. Storm-Versloot L. Verweij C. Lucas J. Ludikhuize J. C. Goslings D. A. Legemate and H. Vermeulen Clinical Relevance of Routinely Measured Vital Signs in Hospitalized Patients: A Systematic Review Journal of Nursing Scholarship 2014 46 39-49
122. W. Montalvo and E. Larson Participant Comprehension of Research for Which They Volunteer: A Systematic Review Journal of Nursing Scholarship 2014 46 423-431
123. S. F. Hawkins and J. Morse The Praxis of Courage as a Foundation for Care Journal of Nursing Scholarship 2014 46 263-270
124. J. Carter S. M. Pouch and E. L. Larson The Relationship Between Emergency Department Crowding and Patient Outcomes: A Systematic Review Journal of Nursing Scholarship 2014 46 106-115
125. K. Massingham S. Fox and A. Smaldone Asthma Therapy in Pediatric Patients: A Systematic Review of Treatment With Montelukast Versus Inhaled Corticosteroids Journal of Pediatric Health Care 2014 28 51-62
126. M. A. Conway C. McCollom and C. Bannon Central Venous Catheter Flushing Recommendations: A Systematic Evidence-Based Practice Review Journal of Pediatric Oncology Nursing 2014 31 185-190
127. Duran S. Siddique and M. Cleary Effects of Leukoreduction and Premedication With Acetaminophen and Diphenhydramine in Minimizing Febrile Nonhemolytic Transfusion Reactions and Allergic Transfusion Reactions During and After Blood Product Administration: A Literature Review With Recommendations for Practice Journal of Pediatric Oncology Nursing 2014 31 223-229
128. Fernbach B. Lockart C. L. Armus L. M. Bashore J. Levine L. Kroon G. Sylvain and C. Rodgers Evidence-Based Recommendations for Fertility Preservation Options for Inclusion in Treatment Protocols for Pediatric and Adolescent Patients Diagnosed With Cancer Journal of Pediatric Oncology Nursing 2014 31 211-222
129. L. Mize N. Harris A. Stokhuyzen T. Avery J. Cash M. Kasse C. Sanborn A. Leonardelli C. Rodgers and M. Hockenberry Neutropenia Precautions for Children Receiving Chemotherapy or Stem Cell Transplantation for Cancer Journal of Pediatric Oncology Nursing 2014 31 200-210
130. Macartney M. B. Harrison E. VanDenKerkhof D. Stacey and P. McCarthy Quality of Life and Symptoms in Pediatric Brain Tumor Survivors A Systematic Review Journal of Pediatric Oncology Nursing 2014 31 65-77
131. Badeaux and D. Hawley A Systematic Review of the Effectiveness of Intravenous Tranexamic Acid Administration in Managing Perioperative Blood Loss in Patients Undergoing Spine Surgery Journal of Perianesthesia Nursing 2014 29 459-465
132. Riddle Intraoperative Brain Activity Monitoring and Postanesthesia Care Unit Length of Stay: Results of a Systematic Review Journal of Perianesthesia Nursing 2014 29 475-479
133. Zhou G. Wang S. F. Yang X. D. He and Y. Liu The Effects of Amino Acid Infusions on Core Body Temperature During the Perioperative Period: A Systematic Review Journal of Perianesthesia Nursing 2014 29 491-500
134. M. Rainforth and M. Laurenson A literature review of Case Formulation to inform Mental Health practice Journal of Psychiatric and Mental Health Nursing 2014 21 206-213
135. Happell C. Platania-Phung and D. Scott A systematic review of nurse physical healthcare for consumers utilizing mental health services Journal of Psychiatric and Mental Health Nursing 2014 21 Nov-22
136. Y. L. Fung Z. Chan and W. T. Chien Role performance of psychiatric nurses in advanced practice: a systematic review of the literature Journal of Psychiatric and Mental Health Nursing 2014 21 698-714
137. S. J. M. Calderon C. A systematic review of oral health behavior research in american adolescents Journal of School Nursing 2014 30 396-403
138. Hildebrand M. Taylor and C. Bradway Elder self-neglect: The failure of coping because of cognitive and functional impairments Journal of the American Association of Nurse Practitioners 2014 26 452-462
139. M. Lefebvre and R. M. John The effect of breastfeeding on childhood overweight and obesity: A systematic review of the literature Journal of the American Association of Nurse Practitioners 2014 26 386-401
140. Butler C. Bowen A. M. Hughes R. Torah I. Ayala J. Tudor and C. D. Metcalf A systematic review of the key factors affecting tissue viability and rehabilitation outcomes of the residual limb in lower extremity traumatic amputees Journal of Tissue Viability 2014 23 81-93
141. W. Ho Acculturation and its implications on parenting for Chinese immigrants: a systematic review Journal of Transcultural Nursing 2014 25 145-58
142. Guffanti Negative Pressure Wound Therapy in the Treatment of Diabetic Foot Ulcers A Systematic Review of the Literature Journal of Wound Ostomy and Continence Nursing 2014 41 233-237
143. P. Garcia-Fernandez P. L. Pancorbo-Hidalgo and J. J. S. Agreda Predictive Capacity of Risk Assessment Scales and Clinical Judgment for Pressure Ulcers Journal of Wound Ostomy and Continence Nursing 2014 41 24-34
144. McGiveron Obesity pregnancy outcomes and caesarean section: a structured review of the combined literature MIDIRS Midwifery Digest 2014 24 35-36
145. N. M. Borg Cunen J./Murray K. A systematic review of midwife-led interventions to address post partum post-traumatic stress Midwifery 2014 30 170-84
146. M. G. Begley M. M./Dencker A./Benstoem C./Berg M./Devane D. Outcome measures in studies on the use of oxytocin for the treatment of delay in labour: a systematic review Midwifery 2014 30 975-82
147. V. D. Smith D./Lundgren I./Eri T./Benstoem C./Devane D. Salutogenically focused outcomes in systematic reviews of intrapartum interventions: a systematic review of systematic reviews Midwifery 2014 30 e151-6
148. T. Fenech G. Tormented by ghosts from their past': a meta-synthesis to explore the psychosocial implications of a traumatic birth on maternal well-being Midwifery 2014 30 185-93
149. Furuta J. Sandall and D. Bick Women's perceptions and experiences of severe maternal morbidity - A synthesis of qualitative studies using a meta-ethnographic approach Midwifery 2014 30 158-169
150. Fillingham S. Peters A. Chisholm and J. Hart Early training in tackling patient obesity: A systematic review of nurse education Nurse Education Today 2014 34 396-404
151. Z. C. Y. Chan Y. T. Chan C. W. Lui H. Z. Yu Y. F. Law K. L. Cheung K. K. Hung S. H. Kei K. H. Yu W. M. Woo and C. T. Lam Gender differences in the academic and clinical performances of undergraduate nursing students: A systematic review Nurse Education Today 2014 34 377-388
152. Andreou E. Papastavrou and A. Merkouris Learning styles and critical thinking relationship in baccalaureate nursing education: A systematic review Nurse Education Today 2014 34 362-371
153. P. Lahtinen H. Leino-Kilpi and L. Salminen Nursing education in the European higher education area - Variations in implementation Nurse Education Today 2014 34 1040-1047
154. R. Al-Dossary P. Kitsantas and P. J. Maddox The impact of residency programs on new nurse graduates' clinical decision-making and leadership skills: A systematic review Nurse Education Today 2014 34 1024-1028
155. Tailakh L. S. Evangelista J. C. Mentes N. A. Pike L. R. Phillips and D. E. Morisky Hypertension prevalence awareness and control in Arab countries: A systematic review Nursing & Health Sciences 2014 16 126-130
156. J. Kesanen H. Leino-Kilpi D. Arifulla M. Siekkinen and K. Valkeapaa Knowledge tests in patient education: A systematic review Nursing & Health Sciences 2014 16 262-273
157. J. Johnstone and S. Turale Nurses' experiences of ethical preparedness for public health emergencies and healthcare disasters: A systematic review of qualitative evidence Nursing & Health Sciences 2014 16 67-77
158. L. Holm and E. Severinsson Surviving depressive ill-health: A qualitative systematic review of older persons' narratives Nursing & Health Sciences 2014 16 131-140
159. E. Kuis G. Hesselink and A. Goossensen Can quality from a care ethical perspective be assessed? A review Nursing Ethics 2014 21 774-793
160. J. C. Manning P. Hemingway and S. A. Redsell Long- term psychosocial impact reported by childhood critical illness survivors: a systematic review Nursing in Critical Care 2014 19 145-156
161. Park and K. L. Schumacher The state of the science of family caregiver-care receiver mutuality: a systematic review Nursing Inquiry 2014 21 140-152
162. S. H. Bae and D. Fabry Assessing the relationships between nurse work hours/overtime and nurse and patient outcomes: Systematic literature review Nursing Outlook 2014 62 138-156
163. Saber Frontline registered nurse job satisfaction and predictors over three decades: A meta-analysis from 1980 to 2009 Nursing Outlook 2014 62 402-414
164. E. Hutchinson L./Stasa H./Jackson D. Deriving consensus on the characteristics of advanced practice nursing: meta-summary of more than 2 decades of research Nursing Research 2014 63 116-28
165. L. Gerchow B. Tagliaferro A. Squires J. Nicholson S. M. Savarimuthu D. Gutnick and M. Jay Latina Food Patterns in the United States A Qualitative Metasynthesis Nursing Research 2014 63 182-193
166. L. McLaughlin and S. Mahon A Meta-Analysis of the Relationship Among Impaired Taste and Treatment Treatment Type and Tumor Site in Head and Neck Cancer Treatment Survivors Oncology Nursing Forum 2014 0 E194-202
167. S. I. S. Mishra R. W./Snyder C./Geigle P./Gotay C. Are exercise programs effective for improving health-related quality of life among cancer survivors? A systematic review and meta-analysis Oncology Nursing Forum 2014 41 E326-42
168. W. M. L. Ling L. Y./So W. K./Chan K. Effects of acupuncture and acupressure on cancer-related fatigue: a systematic review Oncology Nursing Forum 2014 41 581-92
169. P.-J. Oh and S. H. Kim The Effects of Spiritual Interventions in Patients With Cancer: A Meta-Analysis Oncology Nursing Forum 2014 41 E290-301
170. M. A. Hebdon K./McComb S./Sands L. Transitioning patients to survivorship care: a systematic review Oncology Nursing Forum 2014 41 615-25
171. J. Lewis A Systematic Literature Review of the Relationship Between Stretching and Athletic Injury Prevention Orthopaedic Nursing 2014 33 312-320
172. L. C. L.-W. Cole G. Music as an adjuvant therapy in control of pain and symptoms in hospitalized adults: a systematic review Pain Management Nursing 2014 15 406-25
173. L. Quinn L. K. Sheldon and M. E. Cooley Pediatric Pain Assessment by Drawn Faces Scales: A Review Pain Management Nursing 2014 15 909-918
174. Y. W. W. Chen H. H. The effectiveness of acupressure on relieving pain: a systematic review Pain Management Nursing 2014 15 539-50
175. X.-L. Liu J.-Y. Tan T. Wang Q. Zhang M. Zhang L.-Q. Yao and J.-X. Chen Effectiveness of Home-Based Pulmonary Rehabilitation for Patients with Chronic Obstructive Pulmonary Disease: A Meta-Analysis of Randomized Controlled Trials Rehabilitation Nursing 2014 39 36-59
176. Coker J. Ploeg and S. Kaasalainen The Effect of Programs to Improve Oral Hygiene Outcomes for Older Residents in Long-Term Care A Systematic Review Research in Gerontological Nursing 2014 7 87-100
177. Coker J. Ploeg and S. Kaasalainen The Effect of Programs to Improve Oral Hygiene Outcomes for Older Residents in Long-Term Care: A Systematic Review Research in Gerontological Nursing 2014 7 87-100
178. V. L. Beeckman A./Van Hecke A./Verhaeghe S. A systematic review and meta-analysis of incontinence-associated dermatitis incontinence and moisture as risk factors for pressure ulcer development Research in Nursing & Health 2014 37 204-18
179. M. Freitas S. R./Alves T. B./Takahashi J./Kimura A. F. Changes in physiological and behavioral parameters of preterm infants undergoing body hygiene: a systematic review Rev Esc Enferm USP 2014 48 Spec No 178-83
180. M. C. Z. Schveitzer E. L. Role of complementary therapies in the understanding of primary healthcare professionals: a systematic review Rev Esc Enferm USP 2014 48 Spec No 184-91
181. R. Varallo S. D. P. Guimaraes S. A. R. Abjaude and P. D. Mastroianni CAUSES FOR THE UNDERREPORTING OF ADVERSE DRUG EVENTS BY HEALTH PROFESSIONALS: A SYSTEMATIC REVIEW Revista Da Escola De Enfermagem Da Usp 2014 48 739-747
182. T. D. S. Ferreira E. D. Silveira-Lacerda and M. T. A. Garcia-Zapata Genetic counseling for individuals with hemoglobin disorders and for their relatives: a systematic literature review Revista Da Escola De Enfermagem Da Usp 2014 48 928-933
183. J. P. Ribeiro G. C. Gomes and M. B. Thofehrn Health facility environment as humanization strategy care in the pediatric unit: systematic review Revista Da Escola De Enfermagem Da Usp 2014 48 530-539
184. M. E. Cornelio N. M. C. Alexandre and T. M. Sao-Joao Measuring instruments in cardiology adapted into Portuguese language of Brazil: a systematic review Revista Da Escola De Enfermagem Da Usp 2014 48 368-76
185. L. Boss D. H. Kang M. Marcus and N. Bergstrom Endogenous Sex Hormones and Cognitive Function in Older Adults: A Systematic Review Western Journal of Nursing Research 2014 36 388-426
186. S. J. Bahr S. Solverson A. Schlidt D. Hack J. L. Smith and P. Ryan Integrated Literature Review of Postdischarge Telephone Calls Western Journal of Nursing Research 2014 36 84-104
187. V. E. Lyons and L. L. Popejoy Meta-Analysis of Surgical Safety Checklist Effects on Teamwork Communication Morbidity Mortality and Safety Western Journal of Nursing Research 2014 36 245-261
188. Xie and C. Arslanian-Engoren Self-Concepts of Exercise in Frail Older Adults With Heart Failure: A Literature Review Western Journal of Nursing Research 2014 36 1378-1379
189. S. B. Peacock F./McIntyre H. D./Wilkinson S. A review of interventions to prevent Type 2 Diabetes after Gestational Diabetes Women and Birth 2014 27 e7-e15
190. L. Hoang Q./Ogden K. Women's maternity care needs and related service models in rural areas: A comprehensive systematic review of qualitative evidence Women and Birth 2014 27 233-41
191. V. von Sadovszky B. Draudt and S. Boch A Systematic Review of Reviews of Behavioral Interventions to Promote Condom Use Worldviews on Evidence-Based Nursing 2014 11 107-117
192. J. Munday S. Hines K. Wallace A. M. Chang K. Gibbons and P. Yates A Systematic Review of the Effectiveness of Warming Interventions for Women Undergoing Cesarean Section Worldviews on Evidence-Based Nursing 2014 11 383-393
193. M. C. Sawyer and D. E. Nunez Cognitive-Behavioral Therapy for Anxious Children: From Evidence to Practice Worldviews on Evidence-Based Nursing 2014 11 65-71
194. S. M. Breitenstein D. Gross and R. Christophersen Digital Delivery Methods of Parenting Training Interventions: A Systematic Review Worldviews on Evidence-Based Nursing 2014 11 168-176
195. Kuo C. C. Lin and F. M. Tsai Effectiveness of Empowerment-Based Self-Management Interventions on Patients with Chronic Metabolic Diseases: A Systematic Review and Meta-Analysis Worldviews on Evidence-Based Nursing 2014 11 301-315
196. Abdullah D. Rossy J. Ploeg B. Davies K. Higuchi L. Sikora and D. Stacey Measuring the Effectiveness of Mentoring as a Knowledge Translation Intervention for Implementing Empirical Evidence: A Systematic Review Worldviews on Evidence-Based Nursing 2014 11 284-300
197. M. L. Stewart Nutrition Support Protocols and Their Influence on the Delivery of Enteral Nutrition: A Systematic Review Worldviews on Evidence-Based Nursing 2014 11 194-199
198. Griffin K. Watt R. Kimble B. Wallis and L. Shields Systematic Literature Review of Incidence Rates of Low-Speed Vehicle Run-Over Incidents in Children Worldviews on Evidence-Based Nursing 2014 11 98-106
199. H. Lin S. L. Chiang W. C. Tzeng and L. C. Chiang Systematic Review of Impact of Lifestyle-Modification Programs on Metabolic Risks and Patient-Reported Outcomes in Adults With Metabolic Syndrome Worldviews on Evidence-Based Nursing 2014 11 361-368
200. S. Hines M. A. Ramis S. Pike and A. M. Chang The Effectiveness of Psychosocial Interventions for Cognitive Dysfunction in Cancer Patients Who Have Received Chemotherapy: A Systematic Review Worldviews on Evidence-Based Nursing 2014 11 187-193
201. Bursnall The Relationship Between Physical Activity and Depressive Symptoms in Adolescents: A Systematic Review Worldviews on Evidence-Based Nursing 2014 11 376-382
202. Richards V. Coulthard G. Borglin and R. R. Team The State of European Nursing Research: Dead Alive or Chronically Diseased? A Systematic Literature Review Worldviews on Evidence-Based Nursing 2014 11 147-155
